# Risk prediction model for overall survival in lung cancer based on inflammatory and nutritional markers

**DOI:** 10.1038/s41598-025-16443-1

**Published:** 2025-08-22

**Authors:** Hongqi Zhou, Weiyun Jin, Lindi Li, Xiangwen Nie, Weiwei Wu, Ran Chen, Qizhen Xie, Haixia Wu, Weiwei Jiang, Min Tang, Jinhai Wang, Maoyuan Wang

**Affiliations:** 1 Oncology Department, Guiyang Public Health Treatment Center, Guiyang, China; 2 College of Humanities Education, Inner Mongolia Medical University, Hohhot, 010100 China; 3 Medical Records Office, Guiyang Public Health Treatment Center, Guiyang, China; 4 School of Information, Guizhou University of Finance and Economics, Guiyang, China

**Keywords:** Inflammation-Nutrition biomarkers, Lung cancer, Risk prediction model, Healthy aging, Public health, Cancer, Biomarkers, Health care, Medical research, Molecular medicine, Oncology, Pathogenesis, Risk factors

## Abstract

This study aims to develop a multidimensional risk prediction model, identify characteristic inflammation-nutrition biomarkers, and optimize clinical decision-making. The study included 500 lung cancer patients diagnosed between October 2019 and October 2024 at a tertiary medical institution in Guiyang, China. The exposure variables included eight inflammation-nutrition biomarkers: neutrophil-to-lymphocyte ratio (NLR), lymphocyte-to-monocyte ratio (LMR), platelet-to-lymphocyte ratio (PLR), systemic immune-inflammation index (SII), hemoglobin-albumin-lymphocyte-platelet score (HALP), prognostic nutritional index (PNI), hemoglobin-to-red cell distribution width ratio (HRR), and albumin-to-globulin ratio (ALB/GLB). The outcome variable was overall survival (OS). This study aimed to predict 1-year mortality rather than conduct traditional time-to-event survival analysis. All patients were followed until death or a uniform administrative censoring point.LASSO logistic regression was employed to model the outcome as a binary classification (death within 1 year: yes/no).This study employed a small-sample modeling approach, initially using LASSO regression for feature selection and dimensionality reduction, followed by variance inflation factor and collinearity screening for secondary feature selection. Finally, the Support Vector Machine-Recursive Feature Elimination (SVM-RFE) algorithm was used to optimize feature variables. The results showed that age, clinical stage, poor differentiation, ECOG PS 0–1, serum albumin level, LMR, HRR, and ALB/GLB were independent prognostic factors. Based on these factors, a lung cancer mortality risk prediction model was developed, and a corresponding web-based calculator was created, providing a practical tool to support clinical decision-making and personalized treatment strategies.

**Supplementary Information:**

The online version contains supplementary material available at 10.1038/s41598-025-16443-1.

## Introduction

Lung cancer remains the leading malignant tumor worldwide in terms of both incidence and mortality. According to the latest 2024 GLOBOCAN data, approximately 2.5 million new cases of lung cancer are diagnosed globally each year, with 1.8 million deaths attributed to the disease, accounting for 20% of all cancer-related deaths^1^. In China, the five-year survival rate for lung cancer is only about 20%^2^, significantly lower than that of developed countries. With the accelerating pace of population aging, older adults have become the primary demographic affected by lung cancer, with patients aged 65 years and above comprising over 60% of all cases^3^. These epidemiological characteristics not only place greater demands on healthcare services but also pose unique challenges to achieving healthy aging^4^.

Despite advances in modern medicine for the diagnosis and treatment of lung cancer, studies have revealed that even among patients with the same TNM stage, overall survival (OS) can vary significantly due to factors such as inflammation, nutritional status, and comorbidities^5^. Such differences are particularly pronounced in elderly patients and those with chronic infectious diseases^6^. For instance, approximately 15%−20% of lung cancer patients have a history of tuberculosis, a comorbidity that not only significantly reduces the five-year survival rate but also complicates prognostic assessments and diagnostic challenges^7^. Consequently, developing more comprehensive and practical prognostic tools that integrate multifactorial information—especially focusing on inflammation-nutrition status and comorbidities—has become a core focus for both public health and clinical medicine.

In recent years, inflammatory and nutritional markers have garnered widespread attention for their value in cancer prognosis. These markers, which reflect the body’s inflammatory response, immune status, and nutritional condition, provide reliable information for predicting survival outcomes^8^. However, there is a lack of systematic research exploring the comprehensive predictive value of these markers in lung cancer patients. This study incorporates eight multidimensional inflammation-nutrition indicators, including the neutrophil-to-lymphocyte ratio (NLR), lymphocyte-to-monocyte ratio (LMR), platelet-to-lymphocyte ratio (PLR), systemic immune-inflammation index (SII), hemoglobin-albumin-lymphocyte-platelet score (HALP), prognostic nutritional index (PNI), hemoglobin-to-red cell distribution width ratio (HRR), and albumin-to-globulin ratio (ALB/GLB). Unlike traditional studies that focus on a single or limited number of indicators^9–12^this study comprehensively evaluates the systemic impact of inflammation and nutrition, addressing the limitations of previous research. Additionally, determining the optimal cutoff values for these indicators in different patient populations and clinical scenarios holds significant clinical relevance.

To address the aforementioned challenges within the context of integrating healthy aging and public health systems, this study fills a critical research gap. The study has three major innovations: First, it incorporates a comprehensive set of multidimensional inflammatory and nutritional markers, enhancing the model’s predictive power and comprehensiveness. Second, it includes the covariate of concomitant tuberculosis, reflecting the significant impact of public health factors on cancer prognosis. Third, for each inflammatory-nutritional marker, this study provides selectable cutoff values applicable to different clinical scenarios based on patient heterogeneity, marking the first attempt to improve the model’s practical utility and generalizability. By employing rigorous statistical methods, this study aims to develop a more accurate, cost-effective, and broadly applicable predictive model, optimizing clinical decision-making for lung cancer patients and offering robust support for public health initiatives and the promotion of healthy aging.

## Study population

The study was designed as a retrospective cohort study, conducted from October 2019 to October 2024, with follow-up ending on October 31, 2024. The study was conducted at a tertiary hospital in Guiyang.The study population consisted of 520 patients diagnosed with lung cancer. After applying exclusion criteria and accounting for loss to follow-up, a total of 500 lung cancer patients were included in the final analysis, all patients were followed up for ≥ 1 year.The inclusion criteria were as follows: (1) age between 18 and 85 years; (2) histopathologically confirmed diagnosis of lung cancer at TNM stages I to IV; (3) no prior anti-tumor treatment (e.g., surgery, radiotherapy, chemotherapy, targeted therapy, or immunotherapy) before diagnosis; (4) completion of relevant hematological examinations prior to anti-tumor treatment; and (5) availability of complete medical records. The exclusion criteria included: (1) concurrent malignancies of other systems; (2) severe heart, renal, or liver failure; and (3) missing data on exposure variables or outcome variables. All data were extracted from patients’ electronic medical records, including basic demographic information, laboratory test results, and follow-up records. Data collection was performed by trained researchers to ensure accuracy and consistency.

### Exposure variables

The exposure variables in this study included the neutrophil-to-lymphocyte ratio (NLR), platelet-to-lymphocyte ratio (PLR), systemic immune-inflammation index (SII), lymphocyte-to-monocyte ratio (LMR), prognostic nutritional index (PNI), hemoglobin-albumin-lymphocyte-platelet score (HALP), hemoglobin-to-red blood cell distribution width ratio (HRR), and albumin-to-globulin ratio (ALB/GLB). These exposure variables were measured based on blood samples collected at the time of patient admission. All variables were treated as continuous variables. For analytical convenience, some variables were categorized according to clinical relevance or recommendations from the literature. The definitions and calculation methods of these exposure variables strictly adhered to international standards.

## Calculation methods for exposure variables

NLR = Absolute Neutrophil Count(×10⁹/L)/Absolute Lymphocyte Count(×10⁹/L).LMR = Absolute Lymphocyte Count(×10⁹/L)/Absolute Monocyte Count(×10⁹/L). PLR = Platelet Count(×10⁹/L)/Absolute Lymphocyte Count(×10⁹/L).SII = Platelet Count (×10⁹/L)×Absolute Neutrophil Count (×10⁹/L)/Absolute Lymphocyte Count(×10⁹/L).HALP=[Hemoglobin(g/L)×Albumin(g/L)×Absolute Lymphocyte Count(×10⁹/L)]/Platelet Count(×10⁹/L).PNI = Serum Albumin(g/L)+[5×Absolute Lymphocyte Count (×10⁹/L)].HRR = Hemoglobin (g/L)/Red Cell Distribution Width (%). ALB/GLB = Serum Albumin (g/L)/Serum Globulin (g/L).

## Outcome variables

**The outcome variable** was overall survival (OS), defined as the time from the date of diagnosis to the date of death or the last follow-up. The determination of the outcome was based on follow-up records, including information on death or the date of the last follow-up. All survival statuses were independently assessed by researchers using a blinded evaluation method to ensure objectivity and consistency of the results.

## Relevant covariates

The covariates included age (≥ 60 years or < 60 years), sex (male, female), body mass index (BMI; ≥25.5or < 25.5), smoking history (yes/no), pathological type (adenocarcinoma, squamous cell carcinoma, small cell carcinoma, or others), lymph node metastasis (yes/no), distant metastasis (yes/no), clinical stage (stages I–IV), degree of differentiation (poorly differentiated vs. moderately/well differentiated), ECOG performance status score (0–1or ≥ 2), diabetes (yes/no), hypertension (yes/no), and tuberculosis (yes/no). The selection of these covariates was based on prognostic factors reported in previous literature, and all variables were extracted from the patients’ electronic medical records.

## Ethic statement

This study was approved by the Ethics Committee of Guiyang Public Health Treatment Center (Approval No.: [2025] Thesis No. 01). Due to the retrospective nature of the study, Guiyang Public Health Treatment Center Ethics Committee waived the need of obtaining informed consent. The study adhered strictly to the Declaration of Helsinki and relevant ethical guidelines, ensuring full protection of patient privacy. All data used in this research were solely for scientific purposes, and the research team committed to not using patient information for any purposes unrelated to this study.

### Statistical methods

The statistical analysis in this study was conducted in three main phases: data preprocessing, missing data imputation, and feature selection. All analyses were performed using R software (version 3.4.3)0.1. Data Preprocessing: For continuous variables, descriptive statistics and histograms were generated using the DataBook package. Outliers—defined as values exceeding three standard deviations—were treated as missing. Variables with skewed distributions were transformed using the natural logarithm (Ln), and the distributional properties were re-evaluated after transformation. These steps were intended to reduce modeling errors and enhance robustness.For categorical variables, sparse categories were merged based on univariate logistic regression results or clinical reasoning. One-hot encoding was then applied to generate binary indicators, thereby improving model interpretability and predictive performance.2. Missing Data Imputation: The extent and pattern of missingness were first assessed descriptively. Missing values were imputed using the MissForest algorithm, which leverages random forest models to capture complex interactions among variables. To assess imputation quality, the distributions before and after imputation were compared. MissForest has demonstrated strong robustness when applied to mixed-type and high-dimensional datasets.3. Feature Selection and Model Validation: Given the limited sample size, a multi-step feature selection procedure was adopted to minimize the risk of overfitting. The methodological strategy was guided by the BMJ’s recommendations for developing prediction models in datasets with few events.First, LASSO logistic regression was used for dimensionality reduction and preliminary variable selection. Next, variance inflation factor (VIF) analysis was conducted to assess multicollinearity and remove redundant predictors. Finally, recursive feature elimination (RFE) based on a support vector machine (SVM) algorithm was employed to optimize the final feature set.Model performance was evaluated through 500 bootstrap resamples, and calibration was assessed using isotonic regression. All statistical tests were two-sided, and a p-value < 0.05 was considered statistically significant.

## Research technical roadmap

**Fig. 1:**
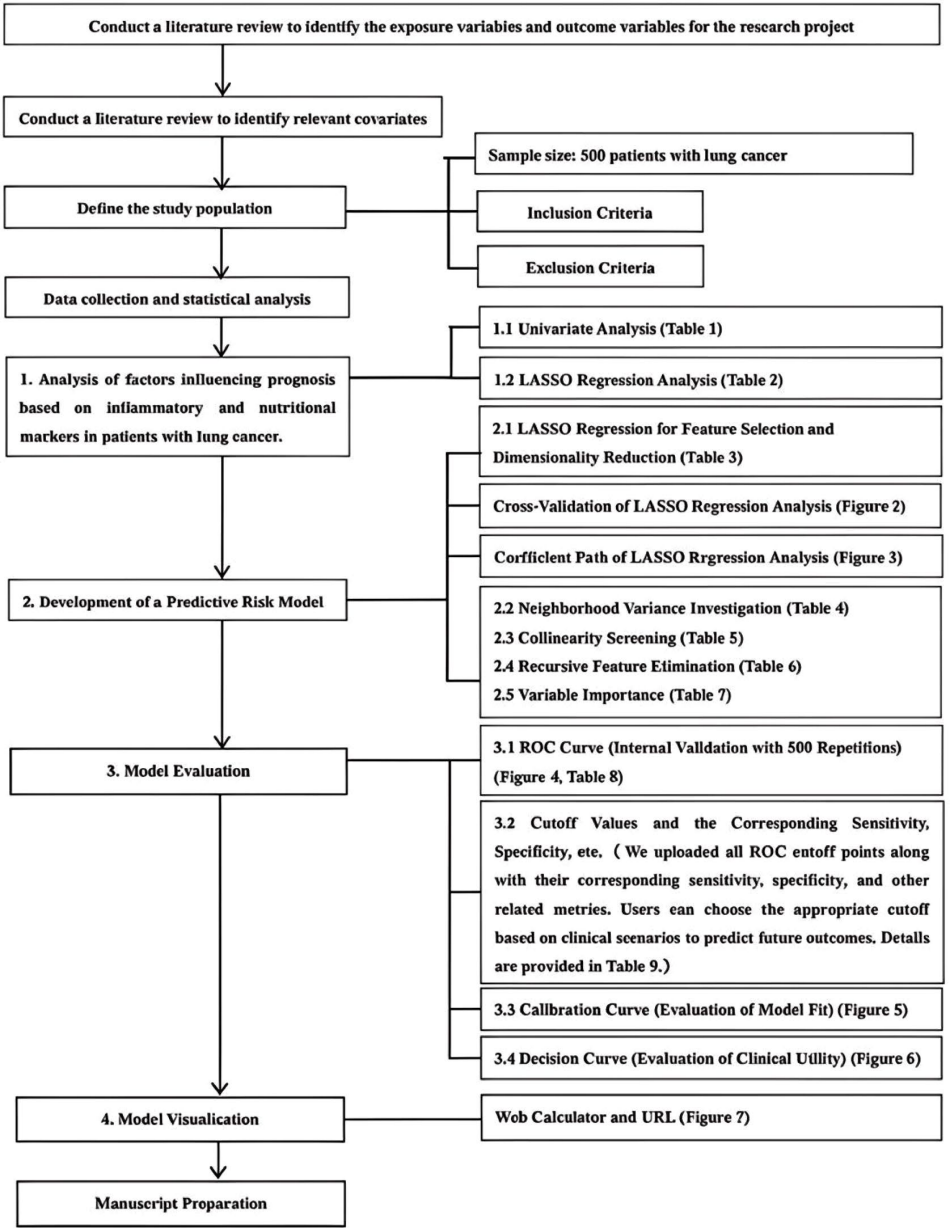
Methodological Framework.

The study primarily investigates four aspects: the analysis of factors influencing prognosis based on inflammatory and nutritional markers in lung cancer patients, the construction of a predictive risk model, model evaluation, and model visualization. For detailed information, please refer to Fig. 1.

## Results

### Analysis of the impact of inflammatory and nutritional markers on prognostic factors in lung cancer patients

#### Univariate analysis

This study ultimately included 500 patients with lung cancer, comprising 178 patients in the survival group and 322 patients in the deceased group. Patients in the survival group had significantly higher levels of lymphocyte count, serum albumin, hemoglobin, prognostic nutritional index (PNI), lymphocyte-to-monocyte ratio (LMR), hemoglobin-to-red cell distribution width ratio (HRR), hemoglobin-albumin-lymphocyte-platelet (HALP) score, albumin-to-globulin ratio (ALB/GLB), as well as a greater proportion of stage I cases and patients with high-to-moderate tumor differentiation. In contrast, patients in the deceased group exhibited significantly higher levels of serum globulin, age, systemic immune-inflammation index (SII Log), platelet-to-lymphocyte ratio (PLR Log), neutrophil-to-lymphocyte ratio (NLR), and a higher proportion of cases with distant metastasis, stage IV disease, Eastern Cooperative Oncology Group performance status (ECOG PS) ≥ 2, and poorly differentiated tumors. Table 1.

**Table 1:** Univariate analysis of clinical and laboratory indicators and poor prognostic factors in lung cancer Patients.

Variable	Survival group (*N* = 178)	Deceased group (*N* = 322)	Standardized difference (95% CI)	*P* value
Laboratory Indicators				
Neutrophil Count (10^9/L)	5.18 ± 2.70	5.66 ± 2.94	0.17 (−0.01, 0.35)	0.071
Platelet Count (10^9/L)	245.18 ± 88.45	263.47 ± 113.03	0.18 (−0.00, 0.36)	0.063
Lymphocyte Count (10^9/L)	1.41 ± 0.51	1.31 ± 0.54	0.20 (0.02, 0.38)	0.034*
Serum Albumin (g/L)	38.24 ± 4.09	35.82 ± 4.87	0.54 (0.35, 0.72)	< 0.001**
Serum Globulin (g/L)	28.51 ± 4.80	30.55 ± 5.85	0.38 (0.20, 0.56)	< 0.001**
Monocyte Count (10^9/L)Log	−0.78 ± 0.45	−0.67 ± 0.50	0.22 (0.04, 0.40)	0.021*
Hemoglobin (g/L)	133.81 ± 18.72	126.96 ± 20.46	0.35 (0.16, 0.53)	< 0.001**
Red Cell Distribution Width (FL)	44.61 ± 27.40	47.55 ± 37.57	0.09 (−0.09, 0.27)	0.360
SII (Log)	6.70 ± 0.82	6.94 ± 0.93	0.28 (0.09, 0.46)	0.004**
PNI	45.30 ± 4.97	42.35 ± 6.01	0.53 (0.35, 0.72)	< 0.001**
LMR	3.26 ± 1.51	2.68 ± 1.42	0.39 (0.21, 0.58)	< 0.001**
PLR Log	5.16 ± 0.52	5.32 ± 0.65	0.27 (0.08, 0.45)	0.006**
HRR	3.14 ± 0.54	2.89 ± 0.60	0.44 (0.25, 0.62)	< 0.001**
NLR	4.40 ± 3.53	5.16 ± 4.12	0.20 (0.01, 0.38)	0.039*
HALP	33.83 ± 20.43	28.30 ± 19.31	0.28 (0.09, 0.46)	0.003**
ALB/GLB	1.38 ± 0.29	1.21 ± 0.29	0.58 (0.39, 0.76)	< 0.001**
**Clinical Characteristics**				
Age (years)	58.28 ± 8.13	64.70 ± 9.84	0.71 (0.52, 0.90)	< 0.001**
BMI	22.54 ± 3.06	22.29 ± 3.38	0.08 (−0.10, 0.26)	0.401
Gender			0.15 (−0.03, 0.33)	0.109
Male	117 (65.73%)	233 (72.59%)		
Female	61 (34.27%)	88 (27.41%)		
Smoking History			0.03 (−0.15, 0.21)	0.735
Yes	116 (65.17%)	214 (66.67%)		
No	62 (34.83%)	107 (33.33%)		
Diabetes			0.02 (−0.16, 0.20)	0.845
Yes	13 (7.30%)	25 (7.79%)		
No	165 (92.70%)	296 (92.21%)		
Hypertension			0.09 (−0.09, 0.28)	0.310
Yes	43 (24.16%)	65 (20.25%)		
No	135 (75.84%)	256 (79.75%)		
Tuberculosis			0.02 (−0.16, 0.20)	0.844
Yes	26 (14.61%)	49 (15.26%)		
No	152 (85.39%)	272 (84.74%)		
Pathological Type			0.23(0.04, 0.41)	0.057
Adenocarcinoma	117 (65.73%)	177 (55.14%)		
Squamous Cell Carcinoma	46 (25.84%)	102 (31.78%)		
Small Cell Carcinoma	15 (8.43%)	42 (13.08%)		
Clinical Stage			0.45(0.26, 0.64)	< 0.001**
Stage I	38 (21.35%)	24 (7.48%)		
Stage II	21 (11.80%)	25 (7.79%)		
Stage III	39 (21.91%)	86 (26.79%)		
Stage IV	80 (44.94%)	186 (57.94%)		
Differentiation			0.37 (0.18, 0.55)	< 0.001**
Low	146 (82.02%)	301 (93.77%)		
Moderate-High	32 (17.98%)	20 (6.23%)		
Distant Metastasis			0.27 (0.09, 0.45)	0.004**
Yes	81 (45.51%)	189 (58.88%)		
No	97 (54.49%)	132 (41.12%)		
ECOG Performance Status			0.51 (0.32, 0.70)	< 0.001**
0–1 score	152 (85.39%)	205 (63.86%)		
≥ 2 score	26 (14.61%)	116 (36.14%)		

Note: 1.*P < 0.05, significant.2.**P < 0.01, highly significant.3.Standardized differences represent effect sizes, with 95% confidence intervals provided in parentheses.

#### LASSO regression analysis and risk prediction formula

In this study, mortality in lung cancer patients was used as the dependent variable. Independent variables included clinical stage (coded as 1 = Stage I, 0 = Stage II/III/IV), differentiation grade (coded as 1 = low differentiation, 0 = moderate/high differentiation), ECOG PS (coded as 1 = ECOG PS 0–1, 0 = ECOG PS ≥ 2), serum albumin (measured value), LMR (measured value), HRR (measured value), ALB/GLB (measured value), and age (measured value).LASSO regression analysis identified ECOG PS 0–1, ALB/GLB, and age as independent prognostic factors for lung cancer. ECOG PS 0–1 and higher ALB/GLB levels were protective factors, while age was a significant risk factor. Specifically, the analysis indicated that each additional year of age increased the risk of mortality by approximately 7%.Table 2. The following formula was provided to calculate the mortality risk prediction score for individual patients: Exp(x) = [−0.94909] + [−0.47464 × 1(Stage I)] + [0.54761 × 1(Low differentiation)] + [−0.85073 × 1(ECOG PS 0–1)] + [−0.00427 × Serum albumin] + [−0.04974 × LMR] + [−0.28638 × HRR] + [−0.98366 × ALB/GLB] + [0.06838 × Age].The probability of mortality is calculated as: Probability = Exp(x)/[1 + Exp(x)].

**Table 2:** LASSO regression analysis of prognostic risk factors in patients with lung Cancer.

Variables	β Coefficient	Standard error	OR (95% CI)	*P* value
(Intercept)	−0.9491	1.3275	−0.7149	0.39
Clinical stage I	−0.4746	0.3251	0.62 (0.33–1.18)	0.144
Poor Differentiation	0.5476	0.3464	1.73 (0.88–3.41)	0.114
ECOG PS 0–1	−0.8507	0.2691	0.43 (0.25–0.72)	0.002*
Serum albumin (g/L)	−0.0043	0.0320	1.00 (0.94–1.06)	0.894
LMR	−0.0497	0.0780	0.95 (0.82–1.11)	0.523
HRR	−0.2864	0.2070	0.75 (0.50–1.13)	0.166
ALB/GLB	−0.9837	0.4663	0.37 (0.15–0.93)	0.035*
Age (year)	0.0684	0.0119	1.07 (1.05–1.10)	< 0.001*

Note: 1.* indicates *P* < 0.05. 2.β Coefficient represents the estimated log odds ratio.

#### Development of a risk prediction model

##### Feature selection using LASSO regression for dimensionality reduction

**Fig. 2:**
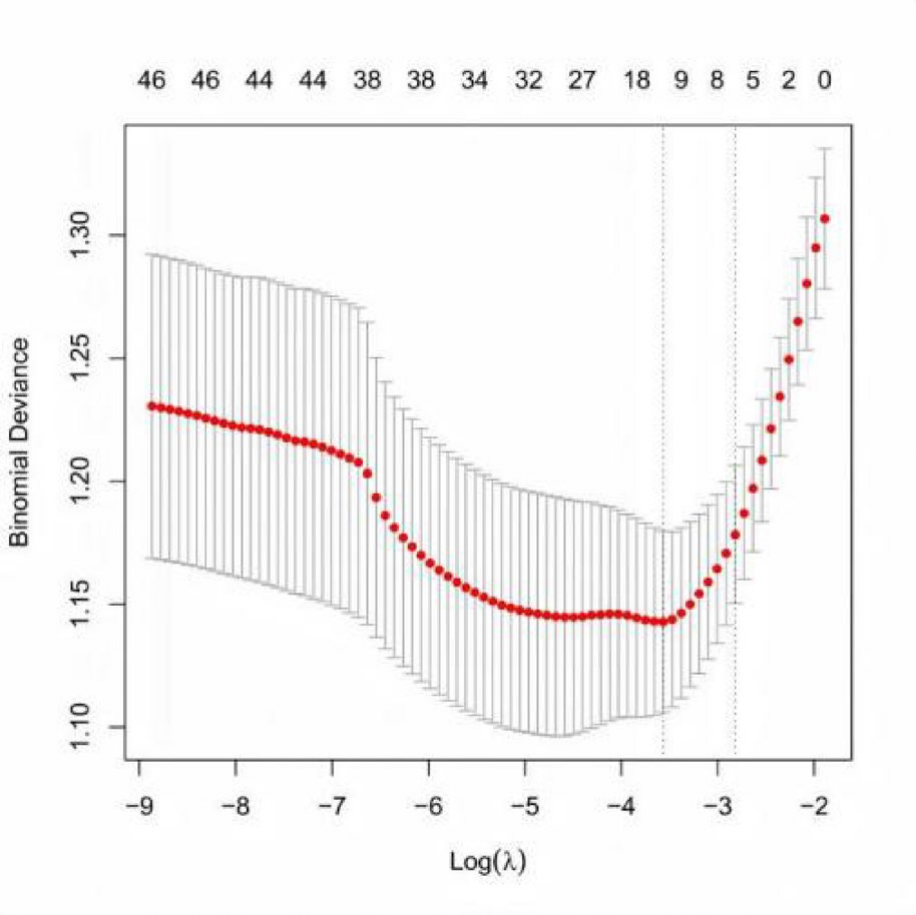
Cross-validation of LASSO regression analysis. Note: The vertical axis represents the cross-validation error, which serves as a metric for assessing model goodness-of-fit. A smaller value indicates better model fit. The lower horizontal axis corresponds to log(λ), where λ is the regularization parameter that controls the complexity of the model. The upper horizontal axis denotes the number of variables retained at different log(λ) values. The two vertical dashed lines indicate the optimal log(λ) value and the log(λ) value within one standard error. Specifically, the upper horizontal axis value corresponding to the left dashed line represents the number of selected variables at the optimal log(λ) value.

**Fig. 3:**
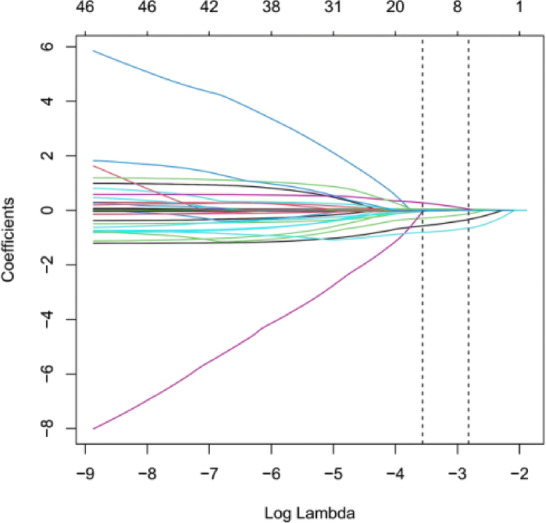
Variable selection path in LASSO regression. Each line in a different color represents a variable. The bottom x-axis corresponds to log(λ) values, while the top x-axis indicates the number of nonzero coefficients (variables) in the model for the respective log(λ) values. The log(λ) value controls the strength of regularization in the model: smaller log(λ) values correspond to weaker regularization, allowing more variables to enter the model, whereas larger log(λ) values correspond to stronger regularization, enhancing the model’s robustness to noise but shrinking many variable coefficients to zero.As the log(λ) value increases (from smaller to larger values), the model complexity decreases, with many variable coefficients gradually shrinking to zero or becoming exactly zero. However, the coefficients of certain variables remain nonzero throughout the process. This observation suggests that the model identifies these variables as more critical features, enabling dimensionality reduction or the selection of the most important predictors.

In this study, LASSO regression analysis with 10-fold cross-validation was used to select the optimal log(λ) values. When log(λ) was − 2.8203 and − 3.5645, the data demonstrated stability and statistical significance. Figure 2 shows the coefficient distribution curve for different log(λ) values. Figure 3 illustrates the stepwise feature selection process, reducing 46 variables to 1. Each curve represents the trajectory of different predictor coefficients as the parameter changes.The optimal eight variables selected for constructing the lung cancer risk prediction model based on inflammation and nutrition markers were: age, Stage I, low differentiation, ECOG PS 0–1, serum albumin, LMR, HRR, and ALB/GLB.Age was identified as an independent risk factor for poor prognosis, with increasing age associated with worse outcomes. Patients with Stage I disease and ECOG PS 0–1 had better prognoses. In contrast, low differentiation, decreased serum albumin levels, and lower ALB/GLB were associated with worse outcomes. Elevated LMR and HRR were linked to improved prognosis.Table 3. Figure 2. Figure 3.

**Table 3:** Multivariate analysis of prognostic factors in two independent Models.

Variables	Model 1		Model 2	
Demographic and Clinical Characteristics	β coefficient	*P* value	β coefficient	*P* value
Age (year)	0.038	< 0.001	0.052	< 0.001
ECOG PS				
0–1	−0.335	< 0.001	−0.571	< 0.001
≥ 2	Reference	-	Reference	-
Clinical Stage				
Stage I	−0.125	< 0.001	−0.293	< 0.001
Stage II-IV	Reference	-	Reference	-
Tumor Differentiation				
Poor	0.040	< 0.001	0.278	< 0.001
Moderate/Well	Reference	-	Reference	-
**Laboratory Parameters**				
Serum Albumin (g/L)	−0.001	0.002	−0.006	< 0.001
ALB/GLB	−0.652	< 0.001	−0.811	< 0.001
LMR	NA	-	−0.040	0.008
HRR	NA	-	−0.047	0.006
**Model Performance**				
Lambda (log)	0.060 (−2.820)	< 0.001	0.028 (−3.565)	< 0.001

Notes:1.β coefficients represent the change in outcome per unit change in the predictor variable.2.P values < 0.001 indicate strong statistical significance.3.Reference categories were selected based on clinical relevance.4.Model 1 and Model 2 represent two independent analytical approaches to validate the findings.5.Variables with no significant association in both models were excluded from the table.

#### Near-Zero variance test of training data

To further refine variable selection and understand their local characteristics and distribution patterns, a near-zero variance test was conducted on multiple variables. The results showed that patient age, ALB/GLB, and ECOG PS scores (≥ 2 and 0–1) were evenly distributed, suggesting their relevance to prognosis. The proportions of LMR, HRR, and ALB/GLB were 93.79%, 94.99%, and 98.20%, respectively, indicating high individual variability among these variables.Table 4.

**Table 4:** Nearest neighbor variance analysis of clinical Variables.

Variables	Freq Ratio	Percent Unique	Distribution Pattern
Demographic Characteristic			
Age (years)	1.26	9.82	Continuous
**Clinical Staging**			
Stage I	7.05	0.40	Categorical
Stage II	9.85	0.40	Categorical
Stage III	2.99	0.40	Categorical
Stage IV	1.14	0.40	Categorical
**Pathological Features**			
Differentiation			
Well/Moderate	8.60	0.40	Categorical
Poor	8.60	0.40	Categorical
**Performance Status**			
ECOG Score			
0–1	2.51	0.40	Categorical
≥ 2	2.51	0.40	Categorical
**Laboratory Parameters**			
Serum Albumin (g/L)	1.00	35.87	Continuous
LMR	1.33	93.79	Continuous
HRR	1.67	94.99	Continuous
ALB/GLB	1.50	98.20	Continuous

#### Collinearity test on training data (Stepwise VIF Selection)

To improve the predictive performance of the model and address irrelevant variables, a collinearity test (VIF) was performed to further refine the feature selection. Variables such as Stage II, moderate/high differentiation, and ECOG PS ≥ 2 were stepwise excluded. The retained variables included Stage I, Stage III, Stage IV, low differentiation, ECOG PS 0–1, serum albumin (g/L), LMR, HRR, ALB/GLB, and age.The analysis identified age, clinical stage, low differentiation, ECOG PS 0–1, serum albumin levels, LMR, HRR, and ALB/GLB as independent prognostic factors.Table 5.

**Table 5:** Variance inflation factors (VIF) analysis through Stepwise variable Selection.

Variables	Step 1	Step 2	Step 3	Step 4
Demographic Characteristics				
Age (years)	1.1	1.1	1.1	1.1
**Clinical Stage**				
Stage I	> 10.0	2.1	2.1	2.1
Stage II	> 10.0	NA	NA	NA
Stage III	> 10.0	3.1	3.1	3.1
Stage IV	> 10.0	4.2	4.2	4.2
**Tumor Differentiation**				
Well/Moderate	> 10.0	> 10.0	NA	NA
Poor	> 10.0	> 10.0	1.3	1.3
**ECOG Performance Status**				
0–1	> 10.0	> 10.0	> 10.0	1.4
≥ 2	> 10.0	> 10.0	> 10.0	NA
**Laboratory Parameters**				
Serum Albumin (g/L)	2.2	2.2	2.2	2.2
LMR	1.3	1.3	1.3	1.3
HRR	1.3	1.3	1.3	1.3
ALB/GLB	1.9	1.9	1.9	1.9

Notes:1.Stepwise variable selection was performed using backward elimination with a VIF threshold of 5.0.2.Variables with VIF > 10.0 were considered to have severe multicollinearity and were candidates for removal.3.Final model (Step 4) achieved optimal variable selection with all VIF values < 5.0.4.Variables removed: Clinical stage (Stage II)、Differentiation grade (Well to moderate differentiation)、ECOG PS(≥ 2)0.5.Predictors used: Clinical stage (Stage I)、Clinical stage (Stage III)、Clinical stage (Stage IV)、Differentiation grade (Poor differentiation)、ECOG PS(0–1)、Serum albumin (g/L)、LMR、HRR、ALB/GLB、Age.

#### Recursive feature elimination

To identify the most relevant feature variables, recursive feature elimination (RFE) was performed. The selected variables included Stage I, low differentiation, ECOG PS 0–1, serum albumin (g/L), LMR, HRR, ALB/GLB, and age.The analysis revealed that LMR had the most significant predictive performance. Serum albumin, ALB/GLB, and HRR also demonstrated strong predictive capabilities. Age, Stage I, low differentiation, and ECOG PS 0–1 showed moderate predictive value.Table 6.

**Table 6:** Analysis of Cross-Validated performance metrics for all Predictors.

Predictor	Accuracy (95% CI)	Kappa (95% CI)	AUC
LMR	0.699 (0.646–0.751)	0.308 (0.189–0.427)	0.731
ALB/GLB	0.695 (0.642–0.748)	0.301 (0.183–0.419)	0.724
Serum albumin	0.693 (0.639–0.747)	0.295 (0.182–0.408)	0.715
ECOG PS	0.685 (0.631–0.739)	0.282 (0.161–0.403)	0.708
Age	0.682 (0.627–0.737)	0.275 (0.147–0.403)	0.701
HRR	0.691 (0.640–0.742)	0.293 (0.181–0.405)	0.712
Clinical stage	0.672 (0.621–0.723)	0.259 (0.138–0.380)	0.694
Differentiation	0.664 (0.608–0.720)	0.236 (0.109–0.363)	0.687

Note: RFE Selected Variables: Clinical Stage (Stage I)、Differentiation Grade (Poor differentiation)、ECOG Performance Status (0–1)、Serum Albumin (g/L)、Lymphocyte-to-Monocyte Ratio (LMR)、High-Risk Ratio (HRR)、Albumin-to-Globulin Ratio (ALB/GLB)、Age (years).

#### Variable importance

Finally, this study systematically evaluated the factors influencing prognosis and identified age as the most critical determinant. The factors ranked in descending order of impact were ECOG PS 0–1, ALB/GLB, poor differentiation, stage I, HRR, LMR, and serum albumin. Table 7.

**Table 7:** Analysis of predictor Importance.

Category	Parameter	Value	Reference Range	*p*-value
**Demographic**	Age (years)	100.00 ± 5.32	N/A	-
**Clinical Assessment**	ECOG Score	0.54 ± 0.12	0–1	< 0.001
**Tumor Characteristics**	Differentiation (Low-grade), n (%)	25.71%	N/A	0.003
	Clinical Stage (Stage I), n (%)	23.56%	N/A	0.002
**Laboratory Parameters**	ALB/GLB Ratio	1.35 ± 0.21	1.2-2.0	0.015
	Serum Albumin (g/L)	35.09 ± 3.42	35–55	0.024
	HRR	22.21 ± 2.15	18–26	0.018
	LMR	8.96 ± 1.03	3.2–9.6	0.013

Notes:1.Values are presented as mean ± SD unless otherwise specified.2.N/A = Not Applicable.3.p-values werecalculated using Student’s t-test for continuous variables and Chi-square test for categorical variables.

#### Model evaluation

##### ROC curve (Internal validation performed 500 Times)

**Fig. 4:**
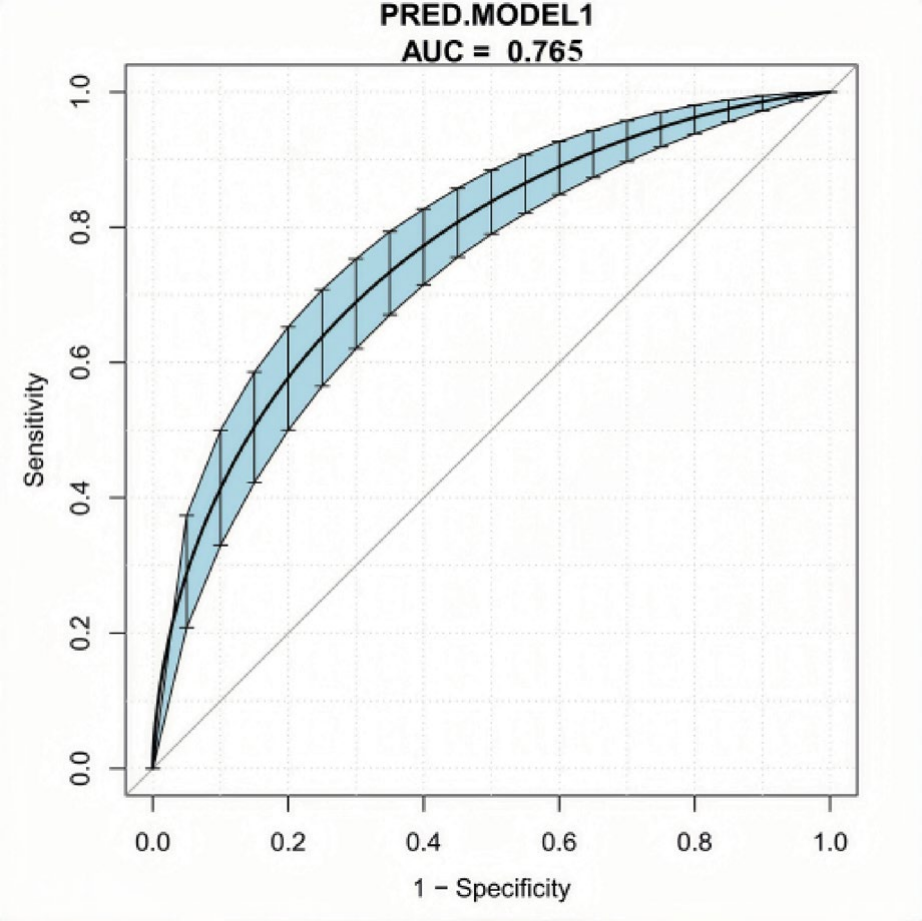
ROC curve.

The performance of the model was evaluated using the ROC curve, with an area under the curve (AUC) of 0.7652 (95% CI: 0.7246–0.8029) and an accuracy of 0.711 (95% CI: 0.669–0.751). The model demonstrated high sensitivity (0.847) and moderate specificity (0.466), indicating good discriminatory power, making it suitable for preliminary disease screening. The positive predictive value (0.741) exceeded the negative predictive value (0.629), suggesting the model is more reliable in identifying positive cases.Internal validation was conducted using 500 bootstrap resamples. Calibration was assessed with isotonic regression fitting. The results showed an F1-score of 0.791 (range: 0–1, with higher values indicating better balance), confirming that the model achieved a good trade-off between precision and recall. These findings highlight the model’s strong predictive accuracy, good sensitivity, and reliable calibration, demonstrating its overall predictive and fitting performance.Table 8. Figure 4.

**Table 8:** Analysis of performance metrics for predictive Models.

Metrics	Value	95% CI
Overall Performance		
Accuracy	0.711	0.669–0.751
AUC	0.765	0.725–0.803
Balanced Accuracy	0.657	-
**Diagnostic Parameters**		
Sensitivity	0.847	-
Specificity	0.466	-
Positive Predictive Value	0.741	-
Negative Predictive Value	0.629	-
**Additional Metrics**		
F1 Score	0.791	-
Detection Rate	0.545	-
Detection Prevalence	0.736	-
Prevalence	0.643	-

Note: ROC = Receiver Operating Characteristic; AUC = Area Under the Curve; PPV = Positive Predictive Value; NPV = Negative Predictive Value. The model was evaluated using the Bootstrap method with 500 internal validations. The model’s accuracy was significantly higher than the null model (0.643, *P* < 0.001). The confusion matrix showed: 83 true negatives, 49 false positives, 95 false negatives, and 272 true positives.

#### Optimal cutoff point

The ROC cut-off points, sensitivity, and specificity for eight inflammation-nutritional markers, including NLR, PLR, SII, LMR, PNI, HALP, HRR, and ALB/GLB, are presented in Supplementary Table S1. Users can select appropriate cut-off points based on their clinical context to predict future outcomes (see Supplementary_Table_S1.pdf).

#### Model calibration

**Fig. 5:**
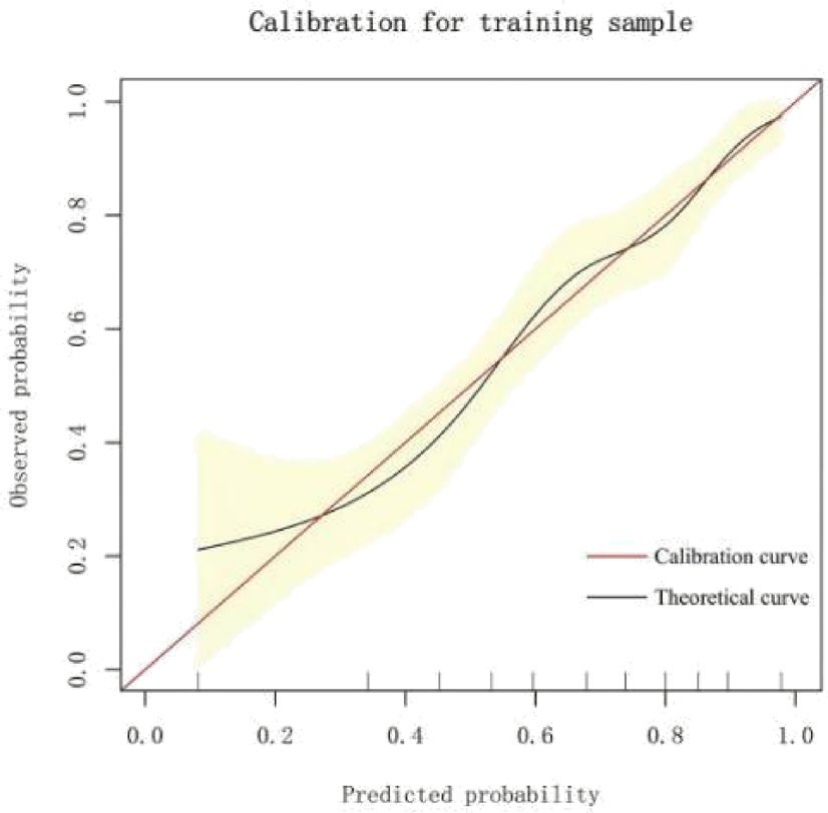
Calibration plot for the predictive model in the training dataset.

The predictive performance of the model was evaluated using a visual calibration curve. The calibration curve closely aligned with the ideal curve, with a slope of 1. This indicates that the risk prediction model has good calibration performance and can provide reliable risk estimates. Figure 5.

##### Clinical utility of the model

**Fig. 6:**
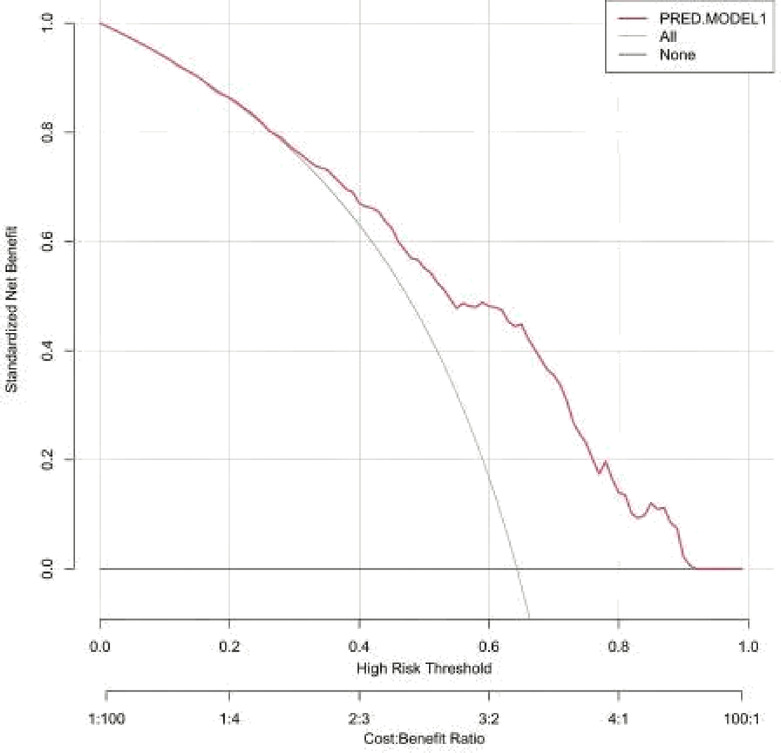
Decision curve analysis of PRED.MODEL1 for clinical decision-making.

The decision curve analysis demonstrated that the predictive model (PRED.MODEL1) maintained stable performance across various risk thresholds. In the low-risk range (0.2–0.3), the model effectively identified high-risk patients requiring intervention while reducing overtreatment. In the moderate-risk range (0.3–0.6), it performed optimally, balancing treatment benefits with potential risks. Even in the high-risk range (0.6–0.8), the model retained good discriminatory ability, aiding in the identification of patients needing aggressive intervention.The model’s curve consistently remained above the reference line and was most prominent in the moderate-risk range (0.3–0.6). These findings indicate that the predictive model can effectively guide clinical decision-making. Figure 6.

#### Web-Based calculator for prognostic risk prediction in patients with lung cancer

**Fig. 7:**
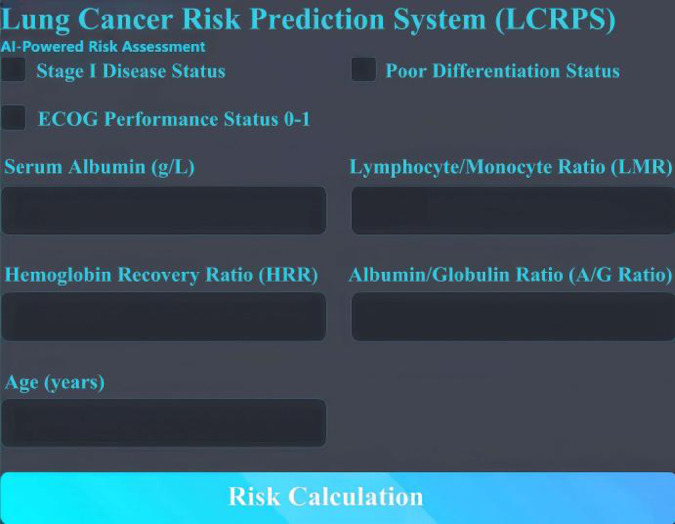
Shows the interface of the web-based calculator.

We have developed a web-based calculator for predicting the prognostic risk of lung cancer patients. Instructions for Use: After accessing Risk Prediction Model for Overall Survival in Lung Cancer Patients, input the patient’s information as follows: Select “Yes” or “No” for “Stage I.“Select “Yes” or “No” for “Poor Differentiation.“Select “Yes” or “No” for “ECOG PS 0–1.“Enter the serum albumin level (g/L) in the designated field.Input the LMR, HRR, and ALB/GLB values in their respective fields.Enter the patient’s age in years.Once all fields are completed, click “Calculate Risk.” The probability of mortality for lung cancer patients will be automatically displayed at the bottom of the interface. For reference, see the webpage calculator interface in Fig. 7.

## Discussion

### Why use LASSO logistic regression

Risk prediction models rely on multiple patient-specific predictors to estimate the probability of clinical events. This study employed a fixed 1-year follow-up window, with all patients achieving definitive follow-up endpoints (death or survival). While Cox regression is the standard approach for time-to-event outcomes, our study utilized a fixed 1-year follow-up period without censoring during the follow-up interval. Moreover, in the context of small sample sizes and high-dimensional variables, LASSO logistic regression can effectively mitigate model overfitting while being well-suited for fixed time point event prediction, making it an appropriate outcome classification method. This approach has been extensively applied in predictive modeling studies.

#### Process of Building the prediction model

To identify the optimal predictive factors, the following steps were undertaken: Univariate Analysis: Each factor was individually examined for its association with the outcome, and statistically significant predictors were preliminarily selected.Stepwise Variable Selection: Stepwise selection was applied to refine the variables. However, given the potential instability of this approach, LASSO regression was utilized to further select variables and achieve dimensionality reduction.Secondary Screening: Key variables were further refined using variance inflation factor analysis and collinearity checks.Final Selection: Recursive feature elimination (RFE) based on a support vector machine (SVM) algorithm was employed for the final selection of predictors.Throughout the process, constraints and shrinkage were applied to the coefficients, ensuring model interpretability and preventing overfitting. Ultimately, predictive factors with strong prognostic value were identified.

### Evaluation of the prediction model

Based on the selected strong predictive factors, the risk prediction model can calculate the probability of a patient experiencing an event. Before applying the model in clinical practice, its predictive value was assessed using the area under the ROC curve (AUC), and internal validation was conducted with 500 bootstrap resamples. Calibration performance was evaluated using isotonic regression fitting. The results showed an F1-score of 0.791 (range: 0–1, with higher values indicating better balance), confirming that the model achieved a good trade-off between precision and recall. This validates the model’s stability and reliability.

### Predictive value of features

This study investigated the association between eight inflammation-nutrition markers (NLR, LMR, PLR, SII, HALP, PNI, HRR, and ALB/GLB) and overall survival (OS) in 500 lung cancer patients. The results identified age, clinical stage, poor differentiation, ECOG PS 0–1, serum albumin levels, LMR, HRR, and ALB/GLB as independent prognostic factors. Among these, age was the most significant independent risk factor, with a 7% increase in mortality risk for every additional year of age. ECOG PS 0–1 and higher ALB/GLB ratios were protective factors for prognosis. Based on these independent factors, a mortality risk prediction model was developed. Elevated LMR, ALB/GLB, and HRR were associated with better prognosis, with LMR showing the strongest predictive performance.

The area under the ROC curve (AUC) for the model was 0.7652 (95% CI: 0.7246–0.8029), with an overall accuracy of 0.711 (95% CI: 0.669–0.751). The model demonstrated high sensitivity (0.8474) and moderate specificity (0.4663). The calibration curve closely aligned with the ideal curve. Decision curve analysis showed that the model remained stable across different risk thresholds, demonstrating excellent clinical utility.

This study provided ROC cut-off points and their corresponding sensitivities and specificities for the eight inflammation-nutrition markers (NLR, PLR, SII, LMR, PNI, HALP, HRR, and ALB/GLB) in various clinical scenarios. Clinicians can select appropriate cut-off points to determine outcomes.

A formula for calculating the mortality risk score for each lung cancer patient was developed: Exp(x) = [−0.94909] + [−0.47464 × 1(Stage I)] + [0.54761 × 1(Poor differentiation)] + [−0.85073 × 1(ECOG PS 0–1)] + [−0.00427 × Serum albumin] + [−0.04974 × LMR] + [−0.28638 × HRR] + [−0.98366 × ALB/GLB] + [0.06838 × Age].The probability of mortality is calculated as: Probability = Exp(x)/[1 + Exp(x)].

A web-based calculator for the lung cancer prognosis risk prediction model was developed, providing a practical tool for clinicians.

## Comparison with existing studies

Our findings align closely with several recent studies while also offering unique insights. For instance, Wei et al.^13^ identified the ALB/GLB ratio as a significant predictor of survival. However, our study has methodological and design advantages. First, we included a broader range of inflammation-nutrition markers and, for the first time, provided clinically applicable cutoff values based on patient heterogeneity, enhancing the model’s generalizability. Second, we employed rigorous variable selection methods, such as LASSO regression and support vector machine recursive feature elimination, to reduce the risk of overfitting. In contrast, Wei et al.‘s study relied primarily on traditional multivariable regression, which may have limitations in feature selection. Additionally, our study incorporated tuberculosis as an important covariate, reflecting the potential influence of public health factors on cancer prognosis, a factor not addressed in Wei et al.‘s work^8^.Thompson et al.^4^ recently highlighted the complex interplay between inflammation markers, the tumor microenvironment, and aging in elderly lung cancer patients. Our observation of a 7% increase in age-related mortality risk is consistent with their findings. However, differences emerged when comparing our results to Li et al.^9^who analyzed the HALP score in solid tumors. While they identified HALP as a strong independent predictor, our study suggests its predictive value is more nuanced when considered alongside other markers. This discrepancy may stem from our more comprehensive approach, which included multiple markers and adjusted for key covariates.Our study also differs from the meta-analysis by Hwang et al.^7^which explored the relationship between tuberculosis and lung cancer risk. While their work involved inflammation markers, their primary focus was on tuberculosis as a risk factor for lung cancer incidence rather than as a predictor of overall survival. Although Hwang et al.‘s study had a larger sample size, their data were pooled from multiple studies, potentially introducing heterogeneity. In contrast, our research utilized single-center patient data, ensuring greater consistency. Another notable difference lies in the analytical methods. Hwang et al. primarily used the traditional Cox proportional hazards model, whereas we combined LASSO regression and ROC curve analysis to validate the predictive performance of our model (AUC = 0.7652), thereby enhancing its reliability and practical utility.

### Strengths and innovations of the study

This study has three primary innovations: Multidimensional Marker Integration: The model incorporated a combination of inflammation-nutrition markers, enhancing its predictive accuracy and clinical applicability.Inclusion of Tuberculosis: The study uniquely analyzed pulmonary tuberculosis, a significant public health factor, highlighting its impact on cancer prognosis.Adaptable Cutoff Values: The model provided selectable cutoff values for different clinical scenarios, increasing its practical application value.In terms of methodology, the study employed LASSO regression for feature selection to achieve dimensionality reduction. Key variables were further refined through variance inflation factor analysis and collinearity checks. Finally, recursive feature elimination using a support vector machine (SVM) algorithm was conducted for the final selection. These steps ensured the inclusion of strong predictive factors, maintained model interpretability, and minimized the risk of overfitting.

### Study limitations

This study has several limitations. First, as a single-center study focusing on patients primarily from Guizhou Province, China, the generalizability of the findings to other populations needs further validation. Second, being an observational study, it can only identify associations between variables rather than establish causal relationships. Finally, despite adjusting for multiple confounders, the potential influence of unmeasured confounding factors cannot be ruled out. Future research should employ multicenter, large-sample prospective studies to confirm these findings.Finally, while traditional survival models such as Kaplan-Meier curves and Cox regression account for censoring and variable follow-up times, this study focuses on 1-year mortality risk prediction. Therefore, logistic regression is methodologically more appropriate, and we acknowledge this distinction as a limitation when comparing to time-dependent survival tools.

#### Significance and future directions

Against the backdrop of accelerated population aging, the burden of chronic disease comorbidities and associated healthcare costs in older adults has become a key global public health challenge. This study focuses on the relationship between inflammation-nutrition markers and lung cancer prognosis, aiming to address the research gap at the intersection of healthy aging and public health systems. By developing a predictive model and an online calculator, the study provides empirical evidence for building integrated healthcare service systems. Future research should further evaluate the applicability of the model in diverse populations and incorporate additional public health factors to create more comprehensive prognostic assessment tools. Moreover, model-based stratification could guide personalized intervention strategies, such as targeted nutritional support or anti-inflammatory treatments. In addition, the exploration of novel biomarkers (e.g., C-reactive protein, interleukin-6, tumor necrosis factor-α) may help enhance the model’s predictive capacity and biological interpretability.

## Conclusion

This study developed and validated a mortality risk prediction model for lung cancer patients by integrating inflammation-nutrition markers. The findings identified age, clinical stage, poor differentiation, ECOG PS 0–1 score, serum albumin level, LMR, HRR, and ALB/GLB as key predictive factors. Additionally, the inclusion of pulmonary tuberculosis as a covariate underscored the significance of public health factors in cancer prognosis.Advanced statistical methods, including LASSO regression, variance inflation factor analysis, collinearity checks, and support vector machine-based feature selection, were employed. The model demonstrated excellent accuracy and generalizability, making it a valuable tool for clinical application.

This study makes an important contribution to the field of personalized medicine by providing a cost-effective and clinically applicable prognostic tool for lung cancer. The web-based calculator developed in this research offers a practical tool for clinicians, aiding in more informed decision-making.

## Supplementary Information

Below is the link to the electronic supplementary material.

Supplementary Material 1

## Data Availability

Data is provided within the manuscript. More data may be provided from corresponding author on request.
