# Supplementary material for: Risk prediction model for overall survival in lung cancer based on inflammatory and nutritional markers
Source: Sci Rep. 2025 Aug 22;15:30840. doi: 10.1038/s41598-025-16443-1 (PMC12373724; doi:10.1038/s41598-025-16443-1)
Supplement: Supplementary file 1 — Supplementary Material 1 [file 41598_2025_16443_MOESM1_ESM.pdf]

Table S1: ROC cut-off values of eight inflammatory and nutritional markers in different clinical scenarios and their corresponding sensitivity, specificity, and related indicators

Case	Cutoff Point	True-positive	False-positive	False-negative	True-negative	Sensitivity	Specificity
1	/	0	0	321	178	0	0.0000
2	0.9775	1	0	320	178	0	0.0031
3	0.9735	2	0	319	178	0	0.0062
4	0.9613	3	0	318	178	0	0.0094
5	0.9575	4	0	317	178	0	0.0125
6	0.9549	5	0	316	178	0	0.0156
7	0.9501	6	0	315	178	0	0.0187
8	0.9499	7	0	314	178	0	0.0218
9	0.9477	8	0	313	178	0	0.0249
10	0.9475	9	0	312	178	0	0.0280
11	0.9461	10	0	311	178	0	0.0312
12	0.9457	11	0	310	178	0	0.0343
13	0.9451	12	0	309	178	0	0.0374
14	0.9439	13	0	308	178	0	0.0405
15	0.9411	14	0	307	178	0	0.0436
16	0.9404	15	0	306	178	0	0.0467
17	0.9393	16	0	305	178	0	0.0498
18	0.9384	17	0	304	178	0	0.0530
19	0.9375	18	0	303	178	0	0.0561
20	0.9357	19	0	302	178	0	0.0592
21	0.9334	20	0	301	178	0	0.0623
22	0.9323	21	0	300	178	0	0.0654
23	0.9317	22	0	299	178	0	0.0685
24	0.9317	23	0	298	178	0	0.0717
25	0.9311	24	0	297	178	0	0.0748
26	0.9264	25	0	296	178	0	0.0779
27	0.9246	26	0	295	178	0	0.0810
28	0.9240	27	0	294	178	0	0.0841

29	0.9235	28	0	293	178	0	0.0872
30	0.9218	29	0	292	178	0	0.0903
31	0.9212	30	0	291	178	0	0.0935
32	0.9209	31	0	290	178	0	0.0966
33	0.9209	31	1	290	177	0.00562	0.0966
34	0.9196	31	2	290	176	0.01124	0.0966
35	0.9178	32	2	289	176	0.01124	0.0997
36	0.9169	33	2	288	176	0.01124	0.1028
37	0.9167	34	2	287	176	0.01124	0.1059
38	0.9158	35	2	286	176	0.01124	0.1090
39	0.9151	36	2	285	176	0.01124	0.1122
40	0.9144	37	2	284	176	0.01124	0.1153
41	0.9116	38	2	283	176	0.01124	0.1184
42	0.9107	39	2	282	176	0.01124	0.1215
43	0.9086	40	2	281	176	0.01124	0.1246
44	0.9083	41	2	280	176	0.01124	0.1277
45	0.9068	42	2	279	176	0.01124	0.1308
46	0.9063	43	2	278	176	0.01124	0.1340
47	0.9051	44	2	277	176	0.01124	0.1371
48	0.9027	45	2	276	176	0.01124	0.1402
49	0.8995	46	2	275	176	0.01124	0.1433
50	0.8986	47	2	274	176	0.01124	0.1464
51	0.8976	48	2	273	176	0.01124	0.1495
52	0.8961	49	2	272	176	0.01124	0.1527
53	0.8957	50	2	271	176	0.01124	0.1558
54	0.8945	51	2	270	176	0.01124	0.1589
55	0.8935	52	2	269	176	0.01124	0.1620
56	0.8934	53	2	268	176	0.01124	0.1651
57	0.8917	54	2	267	176	0.01124	0.1682
58	0.8902	55	2	266	176	0.01124	0.1713
59	0.8901	55	3	266	175	0.01685	0.1713
60	0.8890	56	3	265	175	0.01685	0.1745
61	0.8862	57	3	264	175	0.01685	0.1776

62	0.8861	58	3	263	175	0.01685	0.1807
63	0.8851	59	3	262	175	0.01685	0.1838
64	0.8850	60	3	261	175	0.01685	0.1869
65	0.8840	61	3	260	175	0.01685	0.1900
66	0.8837	62	3	259	175	0.01685	0.1932
67	0.8831	63	3	258	175	0.01685	0.1963
68	0.8793	64	3	257	175	0.01685	0.1994
69	0.8784	65	3	256	175	0.01685	0.2025
70	0.8776	66	3	255	175	0.01685	0.2056
71	0.8763	66	4	255	174	0.02247	0.2056
72	0.8760	67	4	254	174	0.02247	0.2087
73	0.8742	68	4	253	174	0.02247	0.2118
74	0.8736	68	5	253	173	0.02809	0.2118
75	0.8733	69	5	252	173	0.02809	0.2150
76	0.8729	69	6	252	172	0.03371	0.2150
77	0.8724	70	6	251	172	0.03371	0.2181
78	0.8723	71	6	250	172	0.03371	0.2212
79	0.8721	72	6	249	172	0.03371	0.2243
80	0.8703	73	6	248	172	0.03371	0.2274
81	0.8694	74	6	247	172	0.03371	0.2305
82	0.8692	75	6	246	172	0.03371	0.2336
83	0.8666	76	6	245	172	0.03371	0.2368
84	0.8657	77	6	244	172	0.03371	0.2399
85	0.8655	78	6	243	172	0.03371	0.2430
86	0.8645	79	6	242	172	0.03371	0.2461
87	0.8628	80	6	241	172	0.03371	0.2492
88	0.8623	81	6	240	172	0.03371	0.2523
89	0.8622	82	6	239	172	0.03371	0.2555
90	0.8607	82	7	239	171	0.03933	0.2555
91	0.8598	82	8	239	170	0.04494	0.2555
92	0.8598	83	8	238	170	0.04494	0.2586
93	0.8569	84	8	237	170	0.04494	0.2617
94	0.8533	85	8	236	170	0.04494	0.2648

95	0.8533	86	8	235	170	0.04494	0.2679
96	0.8530	87	8	234	170	0.04494	0.2710
97	0.8529	88	8	233	170	0.04494	0.2741
98	0.8528	89	8	232	170	0.04494	0.2773
99	0.8513	89	9	232	169	0.05056	0.2773
100	0.8512	90	9	231	169	0.05056	0.2804
101	0.8508	91	9	230	169	0.05056	0.2835
102	0.8505	92	9	229	169	0.05056	0.2866
103	0.8503	93	9	228	169	0.05056	0.2897
104	0.8502	94	9	227	169	0.05056	0.2928
105	0.8501	95	9	226	169	0.05056	0.2960
106	0.8494	96	9	225	169	0.05056	0.2991
107	0.8493	97	9	224	169	0.05056	0.3022
108	0.8493	97	10	224	168	0.05618	0.3022
109	0.8488	97	11	224	167	0.0618	0.3022
110	0.8483	98	11	223	167	0.0618	0.3053
111	0.8482	99	11	222	167	0.0618	0.3084
112	0.8477	100	11	221	167	0.0618	0.3115
113	0.8464	101	11	220	167	0.0618	0.3146
114	0.8448	101	12	220	166	0.06742	0.3146
115	0.8442	102	12	219	166	0.06742	0.3178
116	0.8419	102	13	219	165	0.07303	0.3178
117	0.8415	103	13	218	165	0.07303	0.3209
118	0.8405	104	13	217	165	0.07303	0.3240
119	0.8404	105	13	216	165	0.07303	0.3271
120	0.8404	105	14	216	164	0.07865	0.3271
121	0.8352	105	15	216	163	0.08427	0.3271
122	0.8335	106	15	215	163	0.08427	0.3302
123	0.8327	107	15	214	163	0.08427	0.3333
124	0.8326	108	15	213	163	0.08427	0.3365
125	0.8325	108	16	213	162	0.08989	0.3365
126	0.8312	108	17	213	161	0.09551	0.3365
127	0.8299	109	17	212	161	0.09551	0.3396

128	0.8299	110	17	211	161	0.09551	0.3427
129	0.8284	111	17	210	161	0.09551	0.3458
130	0.8283	112	17	209	161	0.09551	0.3489
131	0.8250	113	17	208	161	0.09551	0.3520
132	0.8237	114	17	207	161	0.09551	0.3551
133	0.8235	115	17	206	161	0.09551	0.3583
134	0.8202	116	17	205	161	0.09551	0.3614
135	0.8186	116	18	205	160	0.10112	0.3614
136	0.8153	117	18	204	160	0.10112	0.3645
137	0.8140	118	18	203	160	0.10112	0.3676
138	0.8106	118	19	203	159	0.10674	0.3676
139	0.8103	119	19	202	159	0.10674	0.3707
140	0.8089	120	19	201	159	0.10674	0.3738
141	0.8075	121	19	200	159	0.10674	0.3770
142	0.8071	122	19	199	159	0.10674	0.3801
143	0.8071	123	19	198	159	0.10674	0.3832
144	0.8067	124	19	197	159	0.10674	0.3863
145	0.8066	125	19	196	159	0.10674	0.3894
146	0.8060	126	19	195	159	0.10674	0.3925
147	0.8056	126	20	195	158	0.11236	0.3925
148	0.8049	127	20	194	158	0.11236	0.3956
149	0.8033	128	20	193	158	0.11236	0.3988
150	0.8024	129	20	192	158	0.11236	0.4019
151	0.8002	130	20	191	158	0.11236	0.4050
152	0.7995	130	21	191	157	0.11798	0.4050
153	0.7983	131	21	190	157	0.11798	0.4081
154	0.7971	132	21	189	157	0.11798	0.4112
155	0.7971	133	21	188	157	0.11798	0.4143
156	0.7961	134	21	187	157	0.11798	0.4175
157	0.7957	135	21	186	157	0.11798	0.4206
158	0.7935	136	21	185	157	0.11798	0.4237
159	0.7930	136	22	185	156	0.1236	0.4237
160	0.7920	137	22	184	156	0.1236	0.4268

161	0.7912	138	22	183	156	0.1236	0.4299
162	0.7889	139	22	182	156	0.1236	0.4330
163	0.7881	140	22	181	156	0.1236	0.4361
164	0.7874	140	23	181	155	0.12921	0.4361
165	0.7858	140	24	181	154	0.13483	0.4361
166	0.7842	141	24	180	154	0.13483	0.4393
167	0.7829	142	24	179	154	0.13483	0.4424
168	0.7795	143	24	178	154	0.13483	0.4455
169	0.7777	144	24	177	154	0.13483	0.4486
170	0.7767	145	24	176	154	0.13483	0.4517
171	0.7765	145	25	176	153	0.14045	0.4517
172	0.7745	146	25	175	153	0.14045	0.4548
173	0.7718	147	25	174	153	0.14045	0.4579
174	0.7677	148	25	173	153	0.14045	0.4611
175	0.7675	149	25	172	153	0.14045	0.4642
176	0.7669	149	26	172	152	0.14607	0.4642
177	0.7663	150	26	171	152	0.14607	0.4673
178	0.7661	151	26	170	152	0.14607	0.4704
179	0.7661	152	26	169	152	0.14607	0.4735
180	0.7661	153	26	168	152	0.14607	0.4766
181	0.7659	154	26	167	152	0.14607	0.4798
182	0.7640	155	26	166	152	0.14607	0.4829
183	0.7636	156	26	165	152	0.14607	0.4860
184	0.7628	157	26	164	152	0.14607	0.4891
185	0.7625	157	27	164	151	0.15169	0.4891
186	0.7611	158	27	163	151	0.15169	0.4922
187	0.7607	159	27	162	151	0.15169	0.4953
188	0.7575	160	27	161	151	0.15169	0.4984
189	0.7565	160	28	161	150	0.1573	0.4984
190	0.7558	161	28	160	150	0.1573	0.5016
191	0.7555	162	28	159	150	0.1573	0.5047
192	0.7538	162	29	159	149	0.16292	0.5047
193	0.7531	162	30	159	148	0.16854	0.5047

194	0.7516	162	31	159	147	0.17416	0.5047
195	0.7513	163	31	158	147	0.17416	0.5078
196	0.7483	163	32	158	146	0.17978	0.5078
197	0.7467	163	33	158	145	0.18539	0.5078
198	0.7459	164	33	157	145	0.18539	0.5109
199	0.7448	165	33	156	145	0.18539	0.5140
200	0.7399	166	33	155	145	0.18539	0.5171
201	0.7386	167	33	154	145	0.18539	0.5203
202	0.7378	168	33	153	145	0.18539	0.5234
203	0.7367	169	33	152	145	0.18539	0.5265
204	0.7351	170	33	151	145	0.18539	0.5296
205	0.7340	171	33	150	145	0.18539	0.5327
206	0.7337	172	33	149	145	0.18539	0.5358
207	0.7325	172	34	149	144	0.19101	0.5358
208	0.7316	173	34	148	144	0.19101	0.5389
209	0.7314	174	34	147	144	0.19101	0.5421
210	0.7310	175	34	146	144	0.19101	0.5452
211	0.7294	175	35	146	143	0.19663	0.5452
212	0.7290	176	35	145	143	0.19663	0.5483
213	0.7284	177	35	144	143	0.19663	0.5514
214	0.7258	178	35	143	143	0.19663	0.5545
215	0.7247	179	35	142	143	0.19663	0.5576
216	0.7233	180	35	141	143	0.19663	0.5608
217	0.7225	181	35	140	143	0.19663	0.5639
218	0.7206	182	35	139	143	0.19663	0.5670
219	0.7193	183	35	138	143	0.19663	0.5701
220	0.7187	183	36	138	142	0.20225	0.5701
221	0.7183	184	36	137	142	0.20225	0.5732
222	0.7181	185	36	136	142	0.20225	0.5763
223	0.7156	185	37	136	141	0.20787	0.5763
224	0.7119	186	37	135	141	0.20787	0.5794
225	0.7103	187	37	134	141	0.20787	0.5826
226	0.7065	187	38	134	140	0.21348	0.5826

227	0.7048	188	38	133	140	0.21348	0.5857
228	0.7035	189	38	132	140	0.21348	0.5888
229	0.7031	190	38	131	140	0.21348	0.5919
230	0.7029	191	38	130	140	0.21348	0.5950
231	0.7024	192	38	129	140	0.21348	0.5981
232	0.6992	193	38	128	140	0.21348	0.6013
233	0.6978	194	38	127	140	0.21348	0.6044
234	0.6977	195	38	126	140	0.21348	0.6075
235	0.6973	196	38	125	140	0.21348	0.6106
236	0.6969	197	38	124	140	0.21348	0.6137
237	0.6959	198	38	123	140	0.21348	0.6168
238	0.6957	199	38	122	140	0.21348	0.6199
239	0.6957	199	39	122	139	0.2191	0.6199
240	0.6916	200	39	121	139	0.2191	0.6231
241	0.6913	200	40	121	138	0.22472	0.6231
242	0.6899	201	40	120	138	0.22472	0.6262
243	0.6870	202	40	119	138	0.22472	0.6293
244	0.6852	203	40	118	138	0.22472	0.6324
245	0.6850	204	40	117	138	0.22472	0.6355
246	0.6843	205	40	116	138	0.22472	0.6386
247	0.6842	206	40	115	138	0.22472	0.6417
248	0.6827	207	40	114	138	0.22472	0.6449
249	0.6812	208	40	113	138	0.22472	0.6480
250	0.6791	208	41	113	137	0.23034	0.6480
251	0.6791	208	42	113	136	0.23596	0.6480
252	0.6790	209	42	112	136	0.23596	0.6511
253	0.6783	210	42	111	136	0.23596	0.6542
254	0.6752	211	42	110	136	0.23596	0.6573
255	0.6734	212	42	109	136	0.23596	0.6604
256	0.6726	213	42	108	136	0.23596	0.6636
257	0.6662	213	43	108	135	0.24157	0.6636
258	0.6622	214	43	107	135	0.24157	0.6667
259	0.6612	215	43	106	135	0.24157	0.6698

260	0.6602	215	44	106	134	0.24719	0.6698
261	0.6586	216	44	105	134	0.24719	0.6729
262	0.6586	217	44	104	134	0.24719	0.6760
263	0.6576	217	45	104	133	0.25281	0.6760
264	0.6576	218	45	103	133	0.25281	0.6791
265	0.6552	219	45	102	133	0.25281	0.6822
266	0.6531	219	46	102	132	0.25843	0.6822
267	0.6520	220	46	101	132	0.25843	0.6854
268	0.6513	220	47	101	131	0.26404	0.6854
269	0.6511	221	47	100	131	0.26404	0.6885
270	0.6506	222	47	99	131	0.26404	0.6916
271	0.6495	223	47	98	131	0.26404	0.6947
272	0.6484	224	47	97	131	0.26404	0.6978
273	0.6450	225	47	96	131	0.26404	0.7009
274	0.6424	225	48	96	130	0.26966	0.7009
275	0.6417	226	48	95	130	0.26966	0.7041
276	0.6410	227	48	94	130	0.26966	0.7072
277	0.6386	228	48	93	130	0.26966	0.7103
278	0.6380	229	48	92	130	0.26966	0.7134
279	0.6351	230	48	91	130	0.26966	0.7165
280	0.6312	231	48	90	130	0.26966	0.7196
281	0.6289	232	48	89	130	0.26966	0.7227
282	0.6285	233	48	88	130	0.26966	0.7259
283	0.6261	234	48	87	130	0.26966	0.7290
284	0.6254	234	49	87	129	0.27528	0.7290
285	0.6233	235	49	86	129	0.27528	0.7321
286	0.6212	235	50	86	128	0.2809	0.7321
287	0.6195	235	51	86	127	0.28652	0.7321
288	0.6179	235	52	86	126	0.29213	0.7321
289	0.6165	236	52	85	126	0.29213	0.7352
290	0.6153	236	53	85	125	0.29775	0.7352
291	0.6136	237	53	84	125	0.29775	0.7383
292	0.6125	238	53	83	125	0.29775	0.7414

293	0.6121	239	53	82	125	0.29775	0.7446
294	0.6116	239	54	82	124	0.30337	0.7446
295	0.6092	239	55	82	123	0.30899	0.7446
296	0.6059	240	55	81	123	0.30899	0.7477
297	0.6031	241	55	80	123	0.30899	0.7508
298	0.6025	242	55	79	123	0.30899	0.7539
299	0.5993	242	56	79	122	0.31461	0.7539
300	0.5991	242	57	79	121	0.32022	0.7539
301	0.5959	243	57	78	121	0.32022	0.7570
302	0.5878	243	58	78	120	0.32584	0.7570
303	0.5852	243	59	78	119	0.33146	0.7570
304	0.5823	244	59	77	119	0.33146	0.7601
305	0.5815	244	60	77	118	0.33708	0.7601
306	0.5789	245	60	76	118	0.33708	0.7632
307	0.5787	246	60	75	118	0.33708	0.7664
308	0.5783	246	61	75	117	0.3427	0.7664
309	0.5777	246	62	75	116	0.34831	0.7664
310	0.5743	246	63	75	115	0.35393	0.7664
311	0.5743	247	63	74	115	0.35393	0.7695
312	0.5738	247	64	74	114	0.35955	0.7695
313	0.5727	247	65	74	113	0.36517	0.7695
314	0.5721	247	66	74	112	0.37079	0.7695
315	0.5717	247	67	74	111	0.3764	0.7695
316	0.5711	247	68	74	110	0.38202	0.7695
317	0.5664	248	68	73	110	0.38202	0.7726
318	0.5643	248	69	73	109	0.38764	0.7726
319	0.5634	249	69	72	109	0.38764	0.7757
320	0.5621	249	70	72	108	0.39326	0.7757
321	0.5615	250	70	71	108	0.39326	0.7788
322	0.5583	250	71	71	107	0.39888	0.7788
323	0.5581	250	72	71	106	0.40449	0.7788
324	0.5573	250	73	71	105	0.41011	0.7788
325	0.5570	250	74	71	104	0.41573	0.7788

326	0.5566	251	74	70	104	0.41573	0.7819
327	0.5552	252	74	69	104	0.41573	0.7851
328	0.5524	252	75	69	103	0.42135	0.7851
329	0.5517	252	76	69	102	0.42697	0.7851
330	0.5496	253	76	68	102	0.42697	0.7882
331	0.5492	253	77	68	101	0.43258	0.7882
332	0.5491	253	78	68	100	0.4382	0.7882
333	0.5490	254	78	67	100	0.4382	0.7913
334	0.5481	255	78	66	100	0.4382	0.7944
335	0.5476	255	79	66	99	0.44382	0.7944
336	0.5458	255	80	66	98	0.44944	0.7944
337	0.5447	255	81	66	97	0.45506	0.7944
338	0.5447	256	81	65	97	0.45506	0.7975
339	0.5435	256	82	65	96	0.46067	0.7975
340	0.5425	256	83	65	95	0.46629	0.7975
341	0.5417	256	84	65	94	0.47191	0.7975
342	0.5412	256	85	65	93	0.47753	0.7975
343	0.5409	257	85	64	93	0.47753	0.8006
344	0.5378	258	85	63	93	0.47753	0.8037
345	0.5370	259	85	62	93	0.47753	0.8069
346	0.5369	260	85	61	93	0.47753	0.8100
347	0.5351	260	86	61	92	0.48315	0.8100
348	0.5348	260	87	61	91	0.48876	0.8100
349	0.5343	261	87	60	91	0.48876	0.8131
350	0.5328	261	88	60	90	0.49438	0.8131
351	0.5310	262	88	59	90	0.49438	0.8162
352	0.5303	263	88	58	90	0.49438	0.8193
353	0.5299	263	89	58	89	0.5	0.8193
354	0.5291	263	90	58	88	0.50562	0.8193
355	0.5283	264	90	57	88	0.50562	0.8224
356	0.5261	265	90	56	88	0.50562	0.8256
357	0.5253	266	90	55	88	0.50562	0.8287
358	0.5243	266	91	55	87	0.51124	0.8287

359	0.5200	267	91	54	87	0.51124	0.8318
360	0.5141	267	92	54	86	0.51685	0.8318
361	0.5140	268	92	53	86	0.51685	0.8349
362	0.5119	269	92	52	86	0.51685	0.8380
363	0.5117	270	92	51	86	0.51685	0.8411
364	0.5082	270	93	51	85	0.52247	0.8411
365	0.5056	271	93	50	85	0.52247	0.8442
366	0.5042	271	94	50	84	0.52809	0.8442
367	0.5040	272	94	49	84	0.52809	0.8474
368	0.5037	272	95	49	83	0.53371	0.8474
369	0.4972	273	95	48	83	0.53371	0.8505
370	0.4971	274	95	47	83	0.53371	0.8536
371	0.4964	274	96	47	82	0.53933	0.8536
372	0.4964	275	96	46	82	0.53933	0.8567
373	0.4963	275	97	46	81	0.54494	0.8567
374	0.4943	275	98	46	80	0.55056	0.8567
375	0.4939	276	98	45	80	0.55056	0.8598
376	0.4898	276	99	45	79	0.55618	0.8598
377	0.4891	277	99	44	79	0.55618	0.8629
378	0.4877	277	100	44	78	0.5618	0.8629
379	0.4863	277	101	44	77	0.56742	0.8629
380	0.4856	277	102	44	76	0.57303	0.8629
381	0.4837	277	103	44	75	0.57865	0.8629
382	0.4813	278	103	43	75	0.57865	0.8660
383	0.4804	279	103	42	75	0.57865	0.8692
384	0.4766	279	104	42	74	0.58427	0.8692
385	0.4763	280	104	41	74	0.58427	0.8723
386	0.4740	281	104	40	74	0.58427	0.8754
387	0.4727	281	105	40	73	0.58989	0.8754
388	0.4710	281	106	40	72	0.59551	0.8754
389	0.4699	282	106	39	72	0.59551	0.8785
390	0.4694	283	106	38	72	0.59551	0.8816
391	0.4664	283	107	38	71	0.60112	0.8816

392	0.4652	284	107	37	71	0.60112	0.8847
393	0.4641	285	107	36	71	0.60112	0.8879
394	0.4634	286	107	35	71	0.60112	0.8910
395	0.4620	287	107	34	71	0.60112	0.8941
396	0.4595	288	107	33	71	0.60112	0.8972
397	0.4573	288	108	33	70	0.60674	0.8972
398	0.4569	289	108	32	70	0.60674	0.9003
399	0.4556	289	109	32	69	0.61236	0.9003
400	0.4546	290	109	31	69	0.61236	0.9034
401	0.4504	291	109	30	69	0.61236	0.9065
402	0.4493	292	109	29	69	0.61236	0.9097
403	0.4448	293	109	28	69	0.61236	0.9128
404	0.4434	293	110	28	68	0.61798	0.9128
405	0.4414	293	111	28	67	0.6236	0.9128
406	0.4402	293	112	28	66	0.62921	0.9128
407	0.4361	294	112	27	66	0.62921	0.9159
408	0.4353	294	113	27	65	0.63483	0.9159
409	0.4338	294	114	27	64	0.64045	0.9159
410	0.4338	294	115	27	63	0.64607	0.9159
411	0.4334	294	116	27	62	0.65169	0.9159
412	0.4307	294	117	27	61	0.6573	0.9159
413	0.4301	295	117	26	61	0.6573	0.9190
414	0.4266	295	118	26	60	0.66292	0.9190
415	0.4256	295	119	26	59	0.66854	0.9190
416	0.4207	295	120	26	58	0.67416	0.9190
417	0.4136	296	120	25	58	0.67416	0.9221
418	0.4135	297	120	24	58	0.67416	0.9252
419	0.4129	298	120	23	58	0.67416	0.9284
420	0.4126	299	120	22	58	0.67416	0.9315
421	0.4112	299	121	22	57	0.67978	0.9315
422	0.4089	299	122	22	56	0.68539	0.9315
423	0.4079	299	123	22	55	0.69101	0.9315
424	0.4074	300	123	21	55	0.69101	0.9346

425	0.4054	300	124	21	54	0.69663	0.9346
426	0.4038	300	125	21	53	0.70225	0.9346
427	0.4001	301	125	20	53	0.70225	0.9377
428	0.3981	301	126	20	52	0.70787	0.9377
429	0.3970	302	126	19	52	0.70787	0.9408
430	0.3968	302	127	19	51	0.71348	0.9408
431	0.3917	303	127	18	51	0.71348	0.9439
432	0.3916	303	128	18	50	0.7191	0.9439
433	0.3902	303	129	18	49	0.72472	0.9439
434	0.3867	304	129	17	49	0.72472	0.9470
435	0.3858	304	130	17	48	0.73034	0.9470
436	0.3829	304	131	17	47	0.73596	0.9470
437	0.3809	305	131	16	47	0.73596	0.9502
438	0.3803	306	131	15	47	0.73596	0.9533
439	0.3783	306	132	15	46	0.74157	0.9533
440	0.3682	306	133	15	45	0.74719	0.9533
441	0.3667	306	134	15	44	0.75281	0.9533
442	0.3654	306	135	15	43	0.75843	0.9533
443	0.3654	306	136	15	42	0.76404	0.9533
444	0.3648	306	137	15	41	0.76966	0.9533
445	0.3612	306	138	15	40	0.77528	0.9533
446	0.3602	306	139	15	39	0.7809	0.9533
447	0.3517	306	140	15	38	0.78652	0.9533
448	0.3487	307	140	14	38	0.78652	0.9564
449	0.3467	307	141	14	37	0.79213	0.9564
450	0.3425	308	141	13	37	0.79213	0.9595
451	0.3419	308	142	13	36	0.79775	0.9595
452	0.3397	308	143	13	35	0.80337	0.9595
453	0.3314	309	143	12	35	0.80337	0.9626
454	0.3310	309	144	12	34	0.80899	0.9626
455	0.3298	309	145	12	33	0.81461	0.9626
456	0.3295	309	146	12	32	0.82022	0.9626
457	0.3247	310	146	11	32	0.82022	0.9657

458	0.3236	310	147	11	31	0.82584	0.9657
459	0.3232	310	148	11	30	0.83146	0.9657
460	0.3168	311	148	10	30	0.83146	0.9689
461	0.3164	311	149	10	29	0.83708	0.9689
462	0.3139	312	149	9	29	0.83708	0.9720
463	0.3122	312	150	9	28	0.8427	0.9720
464	0.3045	312	151	9	27	0.84831	0.9720
465	0.3038	312	152	9	26	0.85393	0.9720
466	0.2928	312	153	9	25	0.85955	0.9720
467	0.2919	312	154	9	24	0.86517	0.9720
468	0.2858	312	155	9	23	0.87079	0.9720
469	0.2858	313	155	8	23	0.87079	0.9751
470	0.2852	314	155	7	23	0.87079	0.9782
471	0.2713	314	156	7	22	0.8764	0.9782
472	0.2682	315	156	6	22	0.8764	0.9813
473	0.2551	316	156	5	22	0.8764	0.9844
474	0.2548	316	157	5	21	0.88202	0.9844
475	0.2517	316	158	5	20	0.88764	0.9844
476	0.2401	317	158	4	20	0.88764	0.9875
477	0.2333	317	159	4	19	0.89326	0.9875
478	0.2184	317	160	4	18	0.89888	0.9875
479	0.2155	317	161	4	17	0.90449	0.9875
480	0.2148	317	162	4	16	0.91011	0.9875
481	0.2142	317	163	4	15	0.91573	0.9875
482	0.1977	317	164	4	14	0.92135	0.9875
483	0.1951	317	165	4	13	0.92697	0.9875
484	0.1912	317	166	4	12	0.93258	0.9875
485	0.1899	317	167	4	11	0.9382	0.9875
486	0.1802	318	167	3	11	0.9382	0.9907
487	0.1802	318	168	3	10	0.94382	0.9907
488	0.1728	319	168	2	10	0.94382	0.9938
489	0.1525	319	169	2	9	0.94944	0.9938
490	0.1521	319	170	2	8	0.95506	0.9938

491	0.1503	319	171	2	7	0.96067	0.9938
492	0.1492	320	171	1	7	0.96067	0.9969
493	0.1411	320	172	1	6	0.96629	0.9969
494	0.1156	320	173	1	5	0.97191	0.9969
495	0.1130	320	174	1	4	0.97753	0.9969
496	0.1042	320	175	1	3	0.98315	0.9969
497	0.0946	320	176	1	2	0.98876	0.9969
498	0.0927	321	176	0	2	0.98876	1.0000
499	0.0883	321	177	0	1	0.99438	1.0000
500	0.0815	321	178	0	0	1	1.0000

Legend: This table provides the ROC analysis results for eight inflammatory and nutritional markers including NLR, PLR, SII, LMR, PNI, HALP, HRR, and ALB/GLB. The table includes sensitivity, specificity, positive predictive value, negative predictive value, and accuracy under different cut-off values. Clinicians can select appropriate cut-off values based on their specific clinical context to predict patient prognosis.
